# The BDNF/proBDNF ratio as a predictor of antidepressant treatment response in adolescent girls

**DOI:** 10.1192/j.eurpsy.2025.381

**Published:** 2025-08-26

**Authors:** W. Zwolińska, K. Bilska, N. Pytlińska, M. Skibińska, M. Dmitrzak-Węglarz, A. Słopień

**Affiliations:** 1 Child and Adolescent Psychiatry Clinic; 2 Doctoral School; 3 Department of Psychiatric Genetics, Poznan University of Medical Sciences, Poznan, Poland

## Abstract

**Introduction:**

The role of the brain-derived neurotrophic factor (BDNF) in the pathophysiology of depression is well established, with decreased BDNF levels being associated with the emergence of depressive symptoms. More recent studies have reported that the precursor protein – proBDNF – might also be involved in the pathogenesis of depression since both particles are biologically active and elicit opposing effects: mature BDNF promotes the proliferation of neurons and synaptogenesis, while proBDNF evokes neuronal death. The BDNF/proBDNF ratio has been suggested as a possible biomarker of depression state and treatment response among adults with depression. However, no study has analyzed BDNF/proBDNF serum ratio levels in adolescent depressed patients.

**Objectives:**

We aimed to verify the changes in serum BDNF/proBDNF ratio levels during the course of treatment in adolescents with depression in relation to healthy control. We also aimed to investigate whether this parameter could predict the antidepressant treatment outcome.

**Methods:**

Thirty female inpatients, aged 11-17, diagnosed with a first-lifetime depressive episode were assessed at two time-points: before (t0) and after (t1) the minimum six-week period of the first-line antidepressant treatment and compared with thirty age-matched healthy girls. The assessment at t0 and t1 involved the analysis of BDNF and proBDNF serum levels (ELISA method) and clinical symptoms evaluation using standardized depressive symptoms scales: Children’s Depression Inventory (CDI-2) and Hamilton Depression Rating Scale (HDRS). Patients with at least 50% symptom reduction in CDI-2 and HDRS or HDRS<7 were classified as ‘responders.’ The control group underwent one-time BDNF and proBDNF evaluation. The BDNF/proBDNF ratio has been calculated.

**Results:**

The BDNF/proBDNF serum ratio did not significantly differ between the studied and control groups. We proved no significant differences in BDNF/proBDNF serum ratio before and after the antidepressant treatment, regardless of the treatment outcome. However, responders had a significantly higher pretreatment BDNF/proBDNF ratio when compared with non-responders (Figure 1). The ROC analysis revealed that the BDNF/proBDNF ratio at t0 could predict the remission status at t1 with a sensitivity of 66.67% and a specificity of 81.25% (Figure 2).

**Image 1:**

**Figure:**
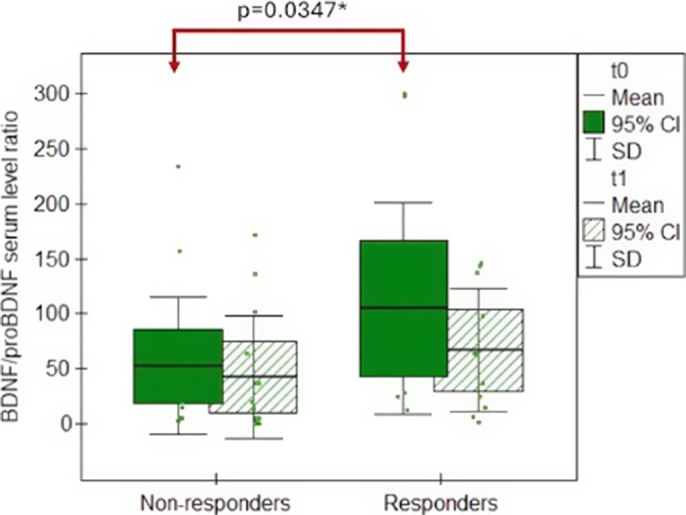

**Image 2:**

**Figure:**
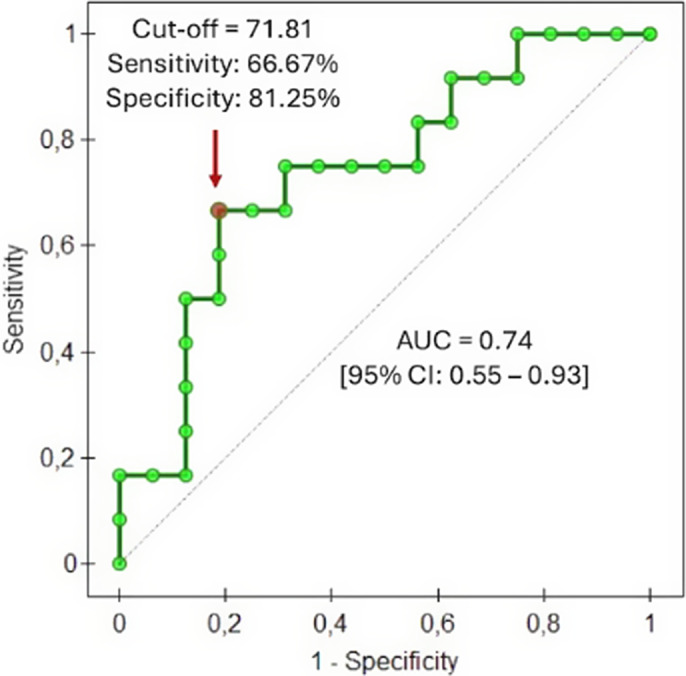

**Conclusions:**

Pretreatment BDNF/proBDNF ratio could be considered a possible biomarker predictive of antidepressant treatment response in adolescent girls.

**Disclosure of Interest:**

None Declared

